# Chitosan nanoparticles for sustained release of metformin and its derived synthetic biopolymer for bone regeneration

**DOI:** 10.3389/fbioe.2023.1169496

**Published:** 2023-07-05

**Authors:** Ning-Xin Chen, Xiao-Lin Su, Yao Feng, Qiong Liu, Li Tan, Hui Yuan, Yun Chen, Jie Zhao, Ya-Qiong Zhao, Marie Aimee Dusenge, Jing Hu, Qin Ye, Ze-Yue Ou-Yang, Meng-Mei Zhong, Qian Zhang, Yue Guo, Yun-Zhi Feng, Yong-Bo Peng

**Affiliations:** ^1^ Department of Stomatology, The Second Xiangya Hospital, Central South University, Changsha, Hunan, China; ^2^ Chongqing Key Laboratory for Pharmaceutical Metabolism Research, The Key Laboratory of Biochemistry and Molecular Pharmacology, College of Pharmacy, Chongqing Medical University, Chongqing, China

**Keywords:** biopolymer, chitosan, metformin, drug release, BMSCs, bone regeneration

## Abstract

**Background:** There are considerable socioeconomic costs associated with bone defects, making regenerative medicine an increasingly attractive option for treating them. Chitosan is a natural biopolymer; it is used in approaches for sustained slow release and osteogenesis, and metformin has osteoinductivity. Our study aimed to synthesize chitosan and human serum albumin (HSA) with a metformin nanoformulation to evaluate the therapeutic effects of this nanoformulation on bone defects *in vitro*.

**Methods:** A pluripotent differentiation assay was performed *in vitro* on mouse bone marrow mesenchymal stem cells (BMSCs). Cell Counting Kit-8 was used to detect whether metformin was toxic to BMSCs. The osteogenesis-related gene expression of osteocalcin (OCN) and osteoprotegerin (OPG) from BMSCs was tested by real-time polymerase chain reaction (PCR). HSA, metformin hydrochloride, and chitosan mixtures were magnetically stirred to finish the assembly of metformin/HSA/chitosan nanoparticles (MHC NPs). The MHC NPs were characterized using transmission electron microscopy (TEM), dynamic light scattering (DLS), and Fourier transform infrared spectroscopy (FT-IR). To test the expression of OCN and OPG, western blot were used. MHC NPs were evaluated *in vitro* for their osteoinductivity using alkaline phosphatase (ALP).

**Results:** BMSCs successfully differentiated into osteogenic and adipogenic lineages *in vitro*. According to real-time PCR, a 50 µM concentration of metformin promoted osteogenesis in BMSCs most effectively by upregulating the osteogenic markers OCN and OPG. The microstructure of MHC NPs was spherical with an average nanosize of 20 ± 4.7 nm and zeta potential of −8.3 mV. A blueshift and redshift were observed in MHC NPs following exposure to wavelengths of 1,600–1,900 and 2,000–3,700 nm, respectively. The encapsulation (%) of metformin was more than 90%. The simulation study showed that MHC NPs have good stability and it could release metformin slowly *in vitro* at room temperature. Upon treatment with the studied MHC NPs for 3 days, ALP was significantly elevated in BMSCs. In addition, the MHC NPs-treated BMSCs upregulated the expression of OPG and OCN, as shown by real-time PCR and western blot.

**Conclusion:** MHC NPs have a stable metformin release effect and osteogenic ability. Therefore, as a derived synthetic biopolymer, it is expected to play a role in bone tissue regeneration.

## 1 Introduction

Bone is a metabolically active supporting tissue (Buck and Dumanian, 2012). There are many causes of bone defects, including trauma, congenital defects, infection, and surgery (Majidinia et al., 2018). The currently available treatments cannot handle the enormous burden of bone defects associated with aging populations. There are limitations in the capability of osteogenic differentiation and the supply of donor organs for transplantation (Adamička et al., 2021; Qasim et al., 2019). Therefore, it is important to find a promising approach to repair bone defects by improving osteogenesis.

Repair methods for bone defects mainly include autologous bone grafts, allograft bone grafts, and biomaterials for tissue engineering (Dimitriou et al., 2011). In terms of bone regeneration for surgery, autogenous bone is considered the gold standard as a result of its osteogenic, osteoconductive, and osteoinductive properties (Galindo-Moreno et al., 2022). Nevertheless, there are a few limitations to autologous bone transplantation, such as limited quantities of autografts available and donor site morbidity (Myeroff and Archdeacon, 2011). However, compared to autologous bones, allografts have a higher risk of infection and immune rejection (Khan et al., 2005). Recent advances in tissue engineering have led to the repair of bone defects using biomaterials, growth factors, and cells (Tang et al., 2016). When used for bone defect repair, biomaterials can overcome immune rejection and pathogenic microorganism infection caused by allograft bone grafts (Khodadadi Yazdi et al., 2020; Zhu et al., 2021). However, bone tissue engineering is limited by the osteogenic properties of biomaterials, and exploring osteogenic bone repair materials is essential (Qian et al., 2019).

Studies on the development of drug delivery systems for bone repair have recently attracted considerable attention by promising to address shortcomings in the treatment of bone diseases and subsequent tissue regeneration (Oliveira É et al., 2021). In previous studies, metformin, an oral diabetes medication, has been found to stimulate osteogenic differentiation of stem cells, thereby enhancing bone formation (Zhao et al., 2020; Bahrambeigi et al., 2019). Metformin activates the AMPK pathway as a means of inducing osteogenic differentiation by regulating the expression of proangiogenic and osteoclastogenic growth factors (Ma et al., 2018). However, the dose-dependent effects of metformin on osteogenesis have been found by some researchers (Ren et al., 2021). Others reported that 500 μM metformin has the strongest effect on osteogenesis, and 250 and 1,000 μM metformin have some increased osteogenic potential compared to the control, but the effect is weaker compared to that of 500 μM metformin. To stimulate bone formation, a stable metformin concentration is essential (Liu et al., 2022).

Currently, there is a rapid expansion of the use of nanotechnology in medicine, specifically in drug delivery (De Jong and Borm, 2008). Several polymeric nanoparticles (NPs) have been studied as promising drug delivery systems in the past few years to improve drug delivery and maintain metformin concentrations (Bhattarai et al., 2006). Among the potentially useful materials investigated, chitosan has gained considerable attention because of its extensive biocompatibility, biodegradability, antimicrobial capacity, and mucoadhesive properties (Peers et al., 2020; Chinnaiyan et al., 2019; Sudhakar et al., 2020). Others have reported that oral delivery of metformin by chitosan nanoparticles has been used for polycystic kidney disease (Wang et al., 2021). Combining metformin with chitosan and pectin provided synergistic antidiabetic effects (Chinnaiyan et al., 2019). A pH-responsive polyelectrolyte complex composed of carboxymethylagarose and chitosan was prepared for dermal drug delivery (Ortiz et al., 2022). It has been found that bioactive molecules can be incorporated into chitosan to accelerate new bone regeneration *in vivo* and improve neovascularization (Aguilar et al., 2019).

In this work, we fabricated a nanocomposite blend consisting of metformin and chitosan for bone tissue engineering. This blend maintains the concentration of metformin at 50 μM. Ionic gelation was used to form the biohybrid nanoparticles. A variety of *in vitro* characterizations, biocompatibility, and enhanced functions for bone tissue regeneration were examined with the optimized formulation. Therefore, metformin/human serum albumin (HSA)/chitosan nanoparticles (MHC NPs) can be used to develop bioactive nanocarrier systems with enhanced functions.

## 2 Materials and methods

### 2.1 Materials

Metformin (commercial grade) was purchased from Solarbio (Beijing, China). Sigma-Aldrich (St. Louis, MO) supplied HSA and phosphotungstic acid. Chitosan powder (Mn ∼ 50,000, deacetylation 90%) was obtained from Haidebei (Jinan, China). Gibco Invitrogen (San Diego, CA, United States) supplied methaemole and fetal bovine serum (FBS), penicillin and streptomycin, and *mycoplasma* antibiotics. Phosphate-buffered saline (PBS) was purchased from Bioss (Beijing, China). Abiowell Biotechnology Co. Ltd. (Changsha, China) provided the Cell Counting Kit-8 (CCK-8). Accurate-Biology (Changsha, China) provided the SteadyPure Quick RNA Extraction Kit, Evo M-MLV RT Premix for qPCR and SYBR^®^ Green Premix Pro Taq HS qPCR Kit. The alkaline phosphatase (ALP) assay kit from Sigma‒Aldrich (Shanghai, China) was used. Oil Red O from Solarbio (Beijing, China) was supplied.

### 2.2 Methods

#### 2.2.1 Preparation of the MHC NPs

**FIGURE 1:**
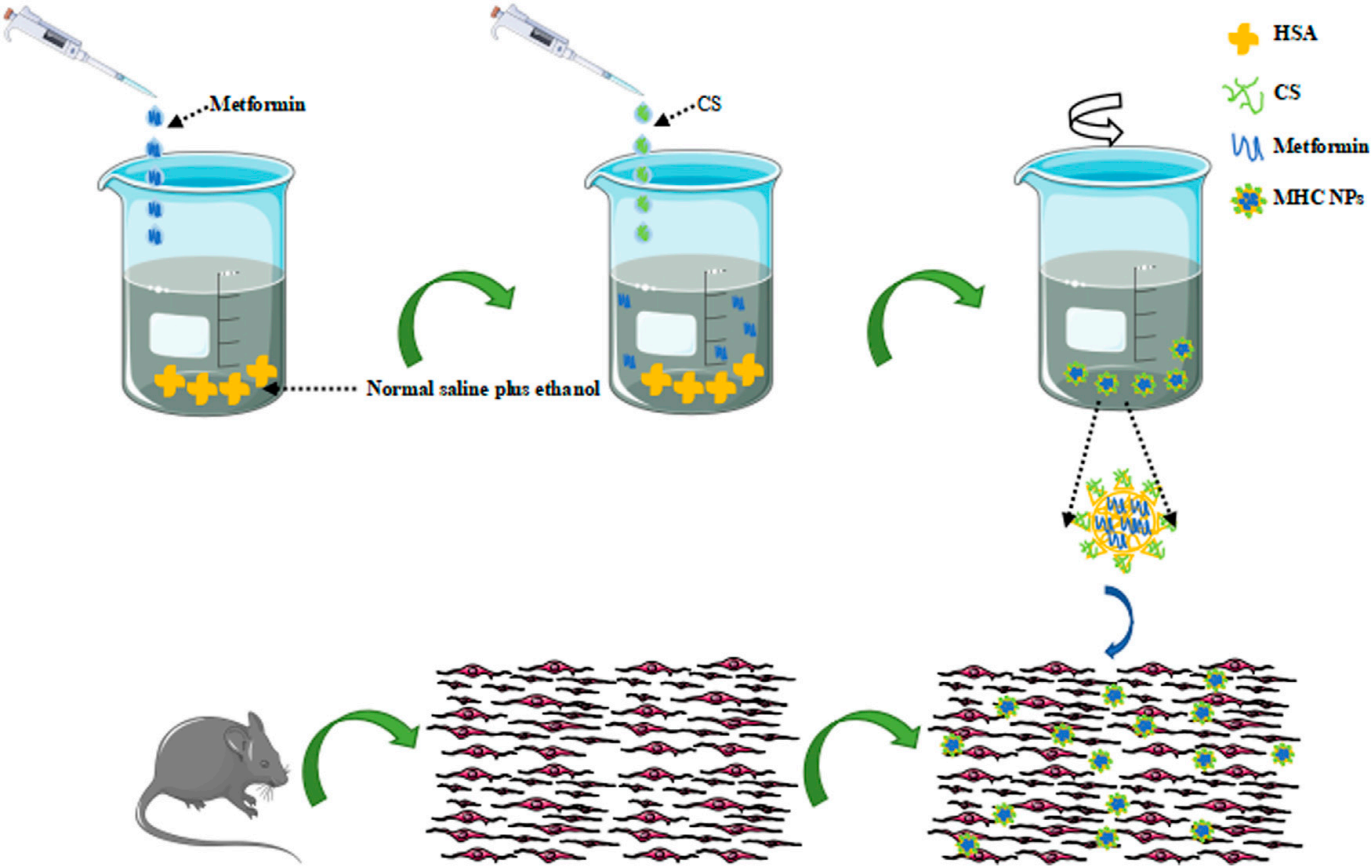
Schematic illustration of MHC NPs. HSA, human serum albumin; CS, chitosan; MHC NPs, metformin/HSA/chitosan nanoparticles. (The sented image of the components’ organization is based on a theorical behaviour of these materials and no empirical data about its real structure has been obtained yet).

HSA (200 mg) was dissolved in 20 mL of normal saline plus 40% ethanol at 60°C by stirring for 90 min. The prepared HSA/ethanol buffer was stirred at 1,500 rpm with a magnetic stirrer for 30 min at 50°C with added metformin hydrochloride (8 mg) and chitosan (30 mg), and the mixture was stirred for 120 min at 37°C with a magnetic stirrer to ensure that the assembly of MHC NPs was completed (Figure 1). The dialysis pockets were filled with normal saline (MWCO 8-14 KD, RT) and kept at 4°C.

#### 2.2.2 TEM

Transmission electron microscopy (TEM, JEM-2100; Japan) was used to observe the morphological characteristics of MHC NPs. Briefly, the diluted MHC NPs solution was dropped onto copper grids and naturally dried, and TEM observation was performed at an accelerating voltage of 200 kV.

#### 2.2.3 DLS

A Zetasizer Nano ZS particle analyzer (Malvern, United Kingdom) was used to evaluate MHC NPs’ stability through dynamic light scattering (DLS).

#### 2.2.4 FT-IR

After each sample was dried and processed into a homogeneous powder with potassium bromide, Fourier-transform infrared spectroscopy (FT-IR, Nicolet IS50, Thermo Fisher Scientific, MA, United States) was used to evaluate the chemical structure of MHC NPs, and the spectra were acquired at 400–4,000 cm^-1^ with a 4 cm^-1^ resolution.

#### 2.2.5 Stability and *in vitro* metformin release evaluation of MHC NPs

The determination of free metformin from MHC NPs in PBS without stirred under room temperature in 20 days was used to evaluate the MHC NPs stability. To analyze the metformin release curve for MHC NPs, 5 mL of MHC NPs was mixed with 10 mL of normal saline (pH 7.4) in a crystal dialysis bag (MWCO 8–14 KD) in 100 mL of normal saline at 37°C at 100 rpm. The samples (300 μL) were collected at set intervals and replaced with normal saline at the same intervals. The free metformin in the released normal saline was measured using high-performance liquid chromatography-electrospray ionization-tandem mass spectrometry (ESI-MS/MS, Agilent G6470A), and the details of the method have been described previously (Ma et al., 2016; Bhujbal and Dash, 2018). High performance liquid chromatography (HPLC) separation was performed on an Agilent C18 column (4.6 × 250 mm, 5 μm) at 37°C, guarded by an Agilent Eclipse XDB-C18 4.6 × 12.5 mm analytical guard column (Agilent, United States). The mobile phase consisted of methanol and water containing 0.1% formic acid (39:61, v/v) at a flow rate of 1 mL/min, and postcolumn splitting (1:4) was used to attain optimal interface flow rates (0.2 mL/min) for MS detection.

#### 2.2.6 Cell culture and treatment

The preparation of mouse bone marrow mesenchymal stem cells (BMSCs) and the induction of osteogenesis have been described previously (Guo et al., 2016). An osteogenic culture medium containing 10 mM glycerophosphate (Sigma), 100 nM dexamethasone (Sigma), and 50 μg/mL L-2-ascorbic acid (Wako Pure Chemical Industries) was used to culture BMSCs. Adipogenic culture medium containing 0.5 mM 3-isobutyl-1-methylxanthine (Sigma), 100 µM indomethacin (Sigma), 10 μg/mL insulin (Abiowell) and 1 µM dexamethasone was used to culture BMSCs.

#### 2.2.7 CCK-8

CCK-8 was used to detect the influence of different concentrations of metformin (0, 1, 5, 10, 50, 100, 200, 500 μM), HSA + chitosan (HSA + CS, 50, 75, 100 μM) and MHC NPs (50, 75, 100 μM) on cell activity on days 1 and 3. Further, 96-well plates were filled with 10 µL of CCK-8 solutions, incubated at 37°C for 2 h, and absorbance at 450 nm was measured using a microplate analyzer (Thermo Scientific, Shanghai, China). Each experiment was repeated at least 3 times.

#### 2.2.8 ALP

A 12-well plate was seeded with BMSCs and incubated at 37°C under 5% CO_2_ for 24 h. Subsequently, the culture medium was replaced with osteogenic medium at different concentrations. Osteogenic medium was treated with different concentrations of metformin (1, 5, 10, 50, 100, 200, 500 μM), HSA + CS (50, 75, 100 μM) and MHC NPs (50, 75, 100 μM). PBS was used several times to wash the plate after 3 days, and 4% paraformaldehyde was used for 30 min to fix the cells. Then, the cells were stained with an ALP assay kit. ALP staining was observed by a cell imaging system.

#### 2.2.9 Oil Red O

A 12-well plate was seeded with BMSCs and incubated at 37°C under 5% CO_2_ for 24 h. Subsequently, the culture medium was replaced with different concentrations of adipogenic medium. The adipogenic medium was replaced every 3 days. After 2–3 weeks of culture, Oil Red O was used to stain lipid droplets, and the cells were observed under a microscope.

#### 2.2.10 Real-time PCR

To measure the expression of osteogenic genes in BMSCs cultured on different concentrations as previously described, real-time polymerase chain reaction (PCR) analysis was performed on the 3rd day. At each time point, after collecting the cells, the SteadyPure Quick RNA Extraction Kit was used to extract the RNA, and complementary DNA (cDNA) was then synthesized using Evo M-MLV RT Premix and a SYBR^®^ Green Premix Pro Taq HS qPCR Kit following the manufacturer’s recommendations. Genes such as osteocalcin (OCN) and osteoprotegerin (OPG) dominated the analysis.

#### 2.2.11 Western blot

The BMSCs were incubated for 3 days in the same manner as described for real-time PCR detection of changes in osteogenic gene expression. Protein was extracted from the cells by digestion, and the concentration of protein was determined by bicinchoninic acid (BCA) analysis. A loading buffer (5 ×) was added at a volume ratio of 4:1, and the protein was boiled for 5 minutes at 100°C to denature it. Subsequently, polyacrylamide gel electrophoresis (SDS-PAGE), membrane transfer, sealing, incubation of primary antibodies against OCN (514636, ZenBioScience), OPG (AB183910, Abcam) and β-ACTIN (AM1021B, Abcepta), incubation, and development of peroxidase-labeled secondary antibodies (AWS0002b, Abiowell) were performed sequentially.

#### 2.2.12 Statistical analysis

For data analysis, SPSS 19.0 (IBM, Armonk, NY, United states) was used. ANOVA was used to analyze the significance of multiple groups. Two groups were compared using an independent *t*-test.

## 3 Results

### 3.1 Osteogenic effects of metformin

**FIGURE 2:**
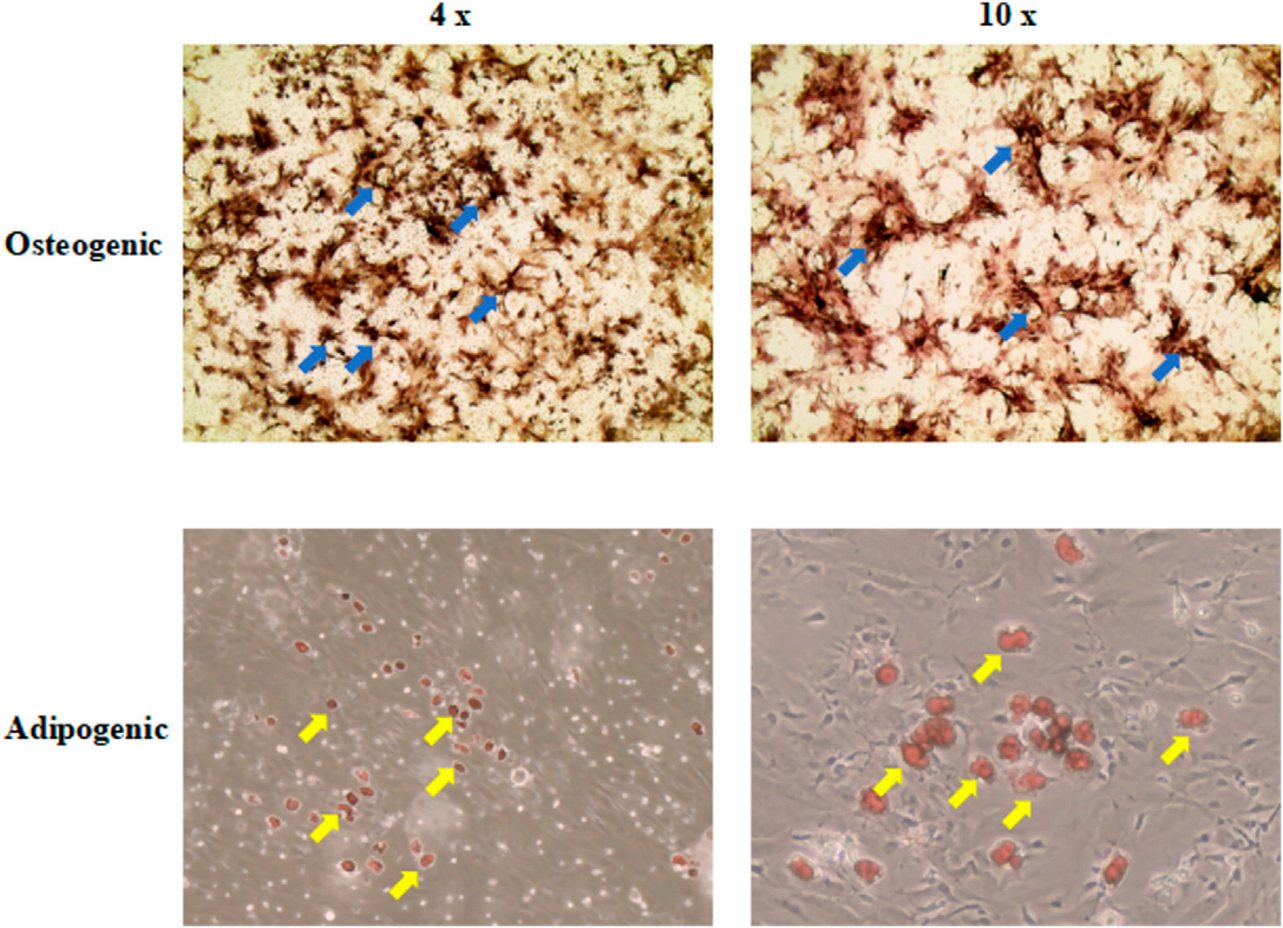
The osteogenic and adipogenic differentiation capacity of BMSCs. ALP and Oil Red O were used to stain calcium nodules and lipid droplets, separately. The upper right corner of each picture showed the results of the control group (4 ×; 10 ×). The blue arrow indicates the calcium nodules. The yellow arrow indicates the lipid droplets. BMSCs, mice bone marrow mesenchymal stem cells; ALP, Alkaline Phosphatase.

Differentiation ability is an important characteristic for stem cell applications in regenerative medicine. After induction in the culture medium, BMSCs exhibited calcium nodules and lipid droplets stained by ALP and Oil Red O (Figure 2). Therefore, BMSCs had osteogenic and adipogenic differentiation capacity.

**FIGURE 3:**
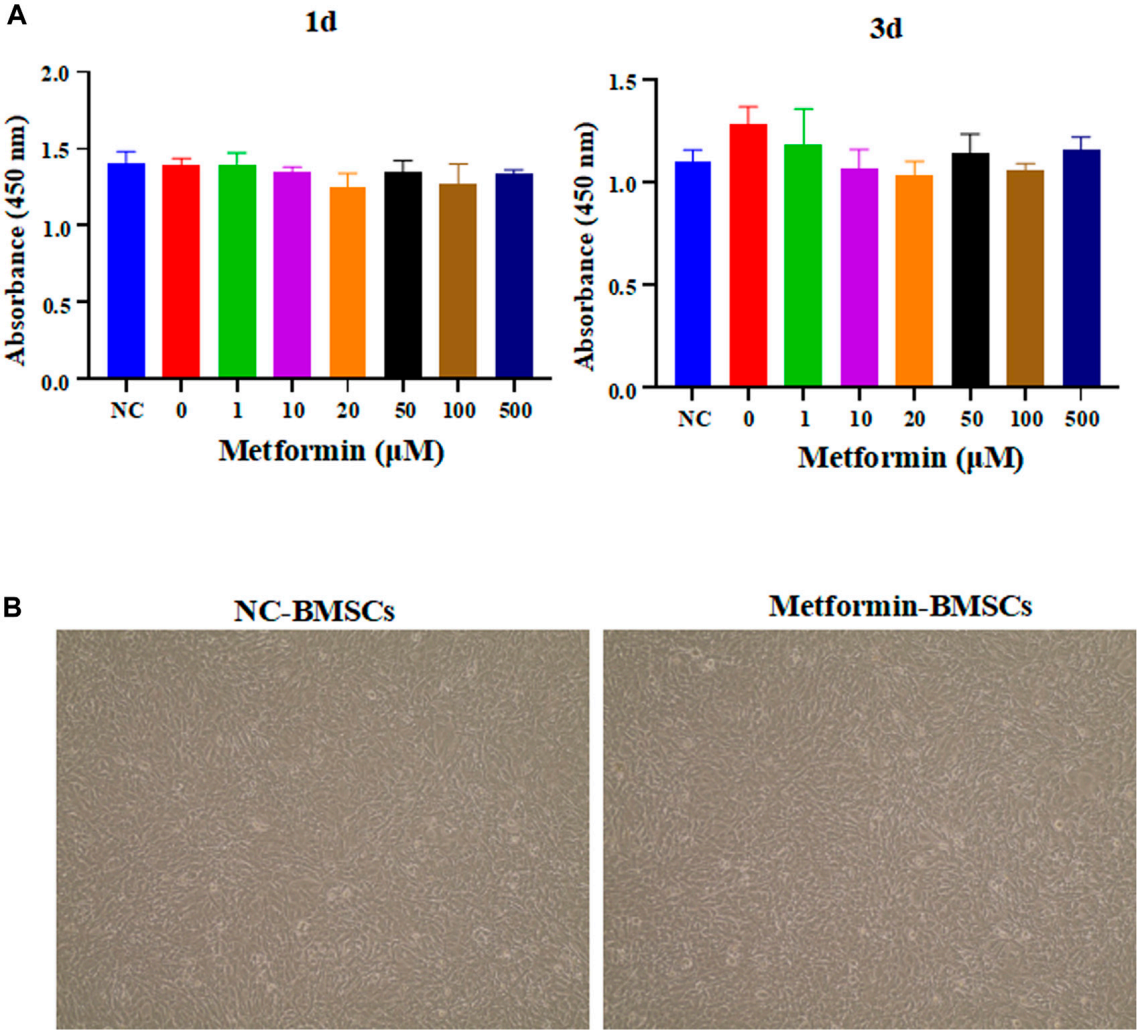
The effect of metformin on the proliferation and morphology of BMSCs. **(A)** The proliferation experiment of metformin on BMSCs. Different concentrations of metformin affect the absorbance of OD450 of BMSCs to be measured by a microplate reader, including 0, 1, 5, 10, 50, 100, 200, 500 μM in 1st day and 3rd day; **(B)** Morphological analysis of stem cells. The cell appearance of the BMSCs and metformin experimental group was observed through an optical microscope (4 ×). BMSCs, bone marrow mesenchymal stem cells.

To determine the optimal concentration of metformin to stimulate cell proliferation in BMSCs, cell proliferation experiments were conducted. According to the results (Figure 3A), metformin was not effective at inhibiting stem cells. Furthermore, the morphological characteristics of BMSCs were not significantly affected by metformin (Figure 3B).

**FIGURE 4:**
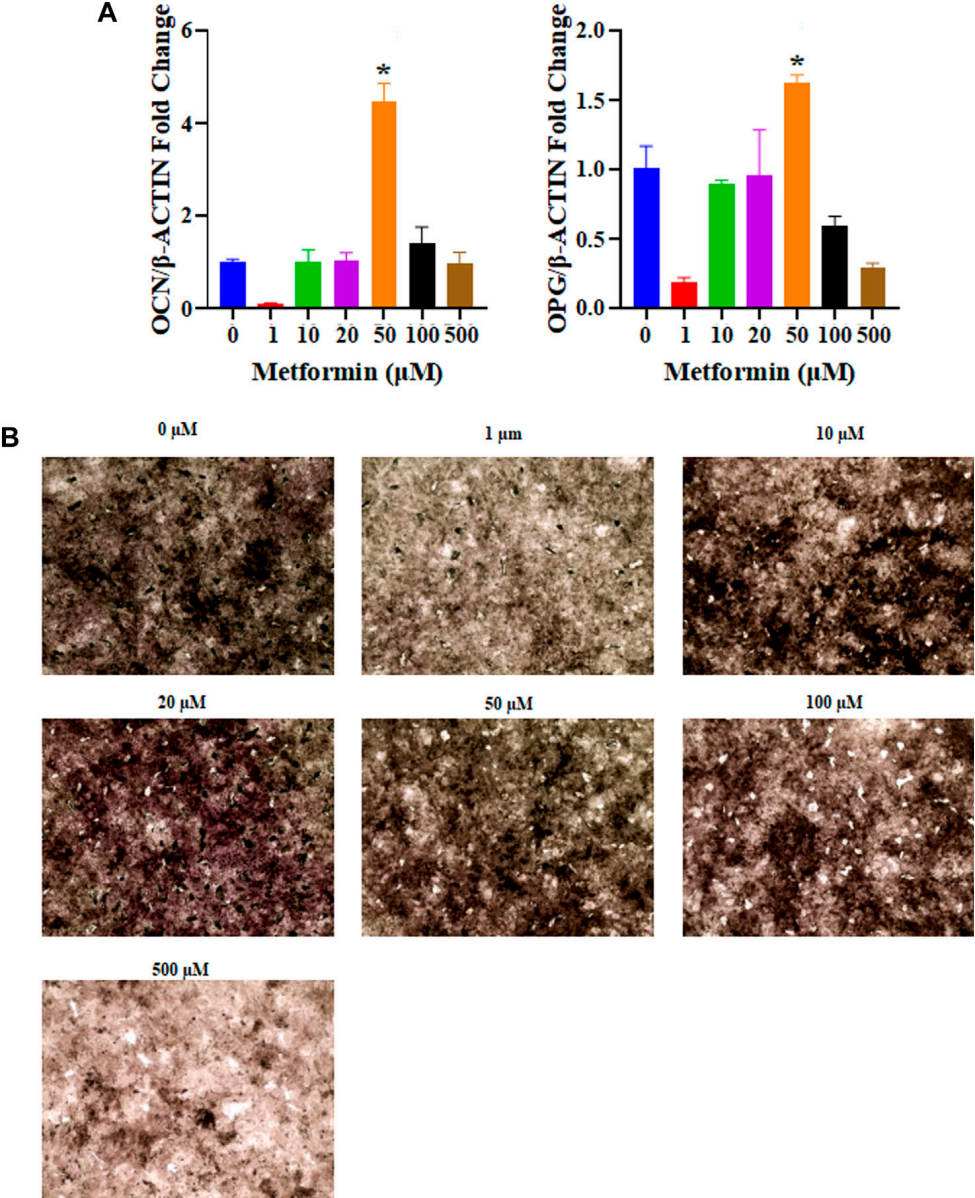
The osteogenic capacity of different concentrations of metformin. **(A)** The relative mRNA expression levels of osteogenic related genes were tested including OCN and OPG. The 50 μM metformin can promote relative mRNA expression levels of OCN and OPG with significant differences compared to other groups (*p* < 0.05); **(B)** Osteogenic induction in metformin at 0, 1, 10, 20, 50, 100, and 500 μM after 3 days, 50 μM of metformin increase ALP expression (black, 4 ×). ALP, Alkaline Phosphatase.

The osteoinductive properties of metformin can promote the differentiation of osteogenic cells. Real-time PCR was used to determine the relative expression of osteogenesis-related genes. According to the obtained results, 50 μM metformin increased the expression of genes related to osteogenic differentiation, including OCN and OPG (*p* < 0.05, Figure 4A). Our study examined the effects of different concentrations of metformin on osteogenesis *in vitro*. The most intense ALP staining occurred under the influence of 50 μM metformin (Figure 4B).

### 3.2 Synthesis and characterization of MHC NPs

**FIGURE 5:**
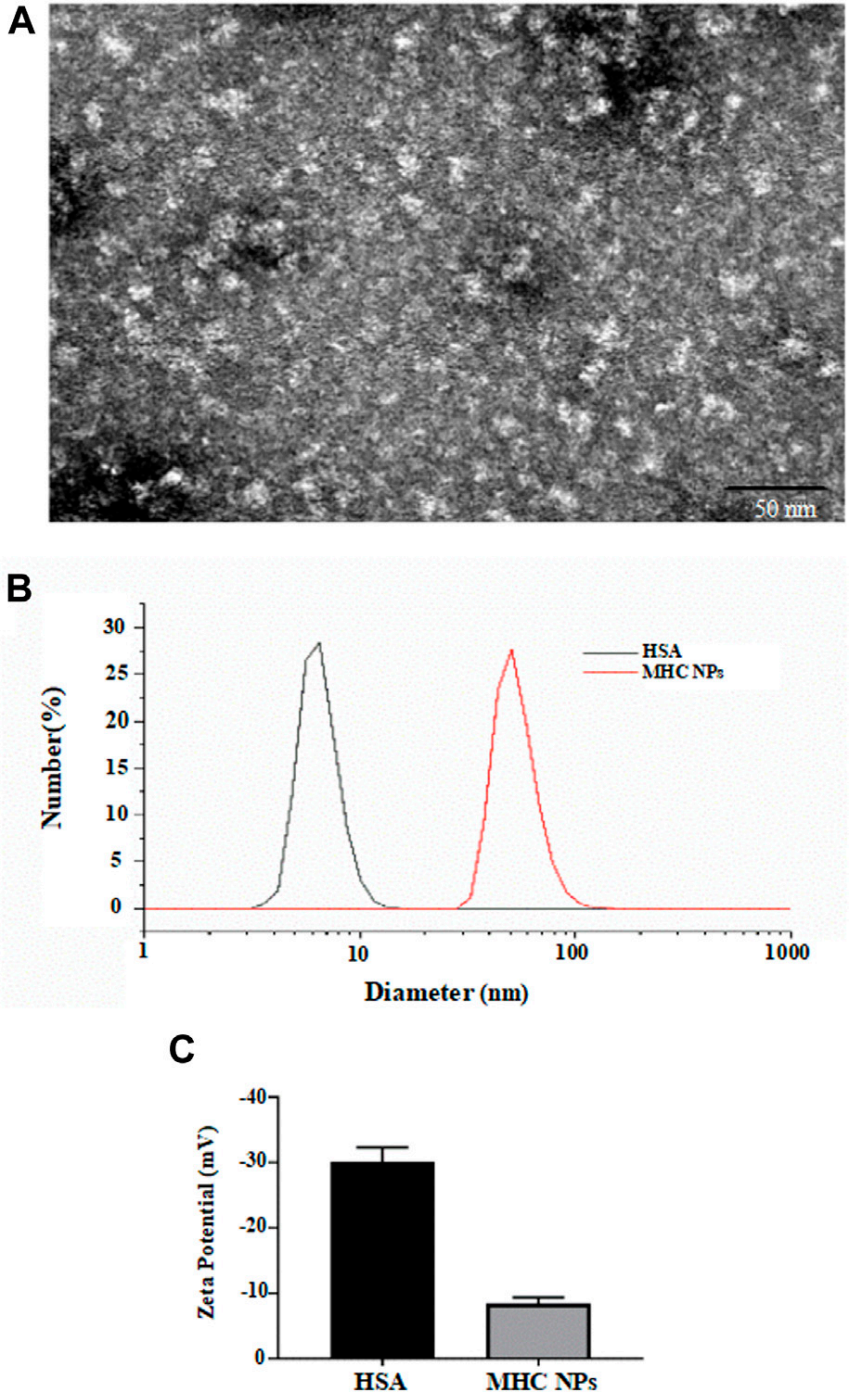
**(A)** Scanning electron microscope image of MHC NPs (Bar = 50 nm). **(B)** Particle size distribution of MHC NPs and HSA. **(C)** Zeta potential of MHC NPs and HSA. HSA, human serum albumin; MHC NPs, metformin/ HSA/chitosan nanoparticles.

As shown in Figure 5A, the microstructure of MHC NPs was spherical with an average size of 20 ± 4.7 nm. The hydrodynamic diameter of MHC NPs (50 ± 9.8 nm) was larger than that of HSA (7 ± 2.1 nm) alone (Figure 5B). The ζ-potential of MHC NPs was less negative than that of HSA (−8.3 mV vs. −30.0 mV; Figure 5C) due to the positive charge of chitosan in deionized water, which may improve cell uptake. The structures and compositions of MHC NPs were determined using FT-IR analysis. A blueshift and redshift were observed in MHC NPs following exposure to 1,600–1,900 and 2,000–3,700 nm wavelengths than HSA or chitosan, respectively, potentially due to the formation of MHC NPs. The characteristic peak of metformin was not obvious. It is because that MHC NPs forms were complex, and most of them were HSA and chitosan (Supplementary Figure S1). Furthermore, the encapsulation efficiency (%) of metformin in MHC NPs was 90% ± 4.35% (w/w) by the reported HPLC-MS/MS method (Ma et al., 2016; Bhujbal and Dash, 2018), and the drug loading (%) was 3.05 % ± 0.14% (*w/w*).

### 3.3 Stability and release performance of MHC NPs *in vitro*

**FIGURE 6:**
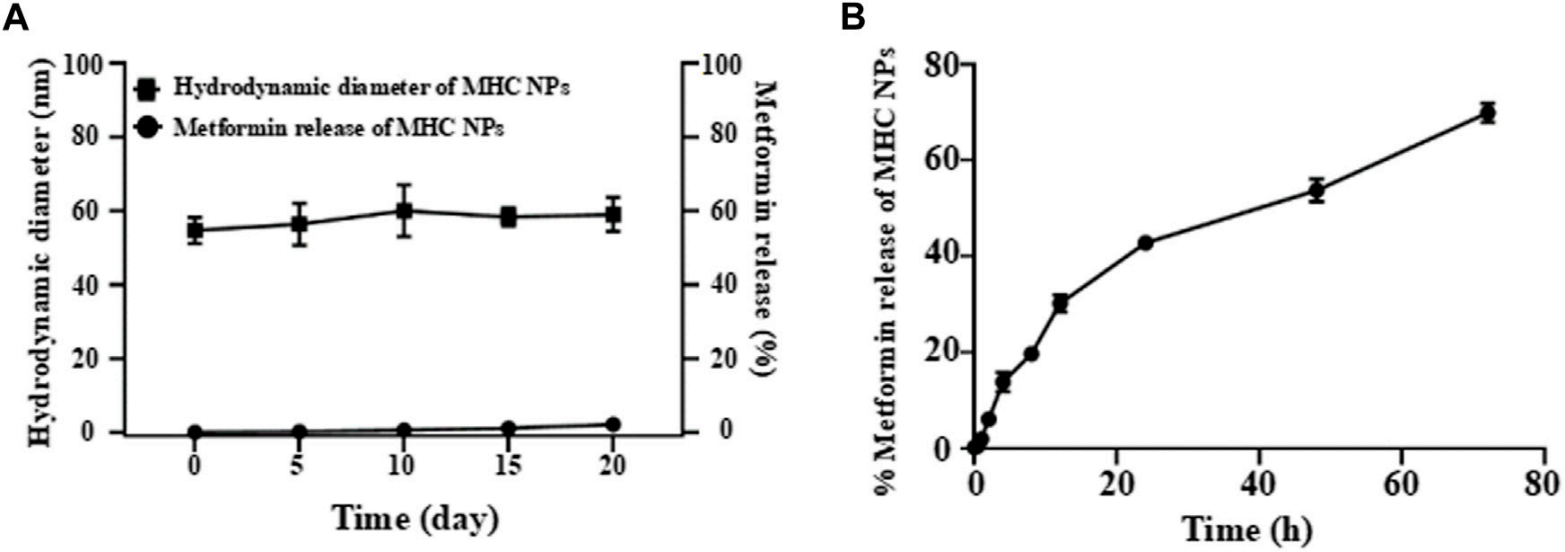
**(A)** The free metformin release from MHC NPs at room temperature under normal saline in 20 d. **(B)** The *in vitro* metformin release profile from MHC NPs. HSA: human serum albumin. MHC NPs: metformin/HSA/chitosan nanoparticles.

The obtained free metformin release results showed that MHC NPs exhibited good stability over 20 days at room temperature (Figure 6A), indicating MHC NPs can keep stable under normalisotonic solution. The *in vitro* metformin release profile from MHC NPs is shown in Figure 6B, we can observe that the MHC NPs exhibited the slow percent cumulative release (< 40%) in the 12 h at 37°C. This slow release may be because of the positive-negative charge interaction between metformin/chitosan and HSA, and denature/renature crosslink of HSA by the changed content of ethanol, which induced the MHC NPs form through relatively strong binding.

**FIGURE 7:**
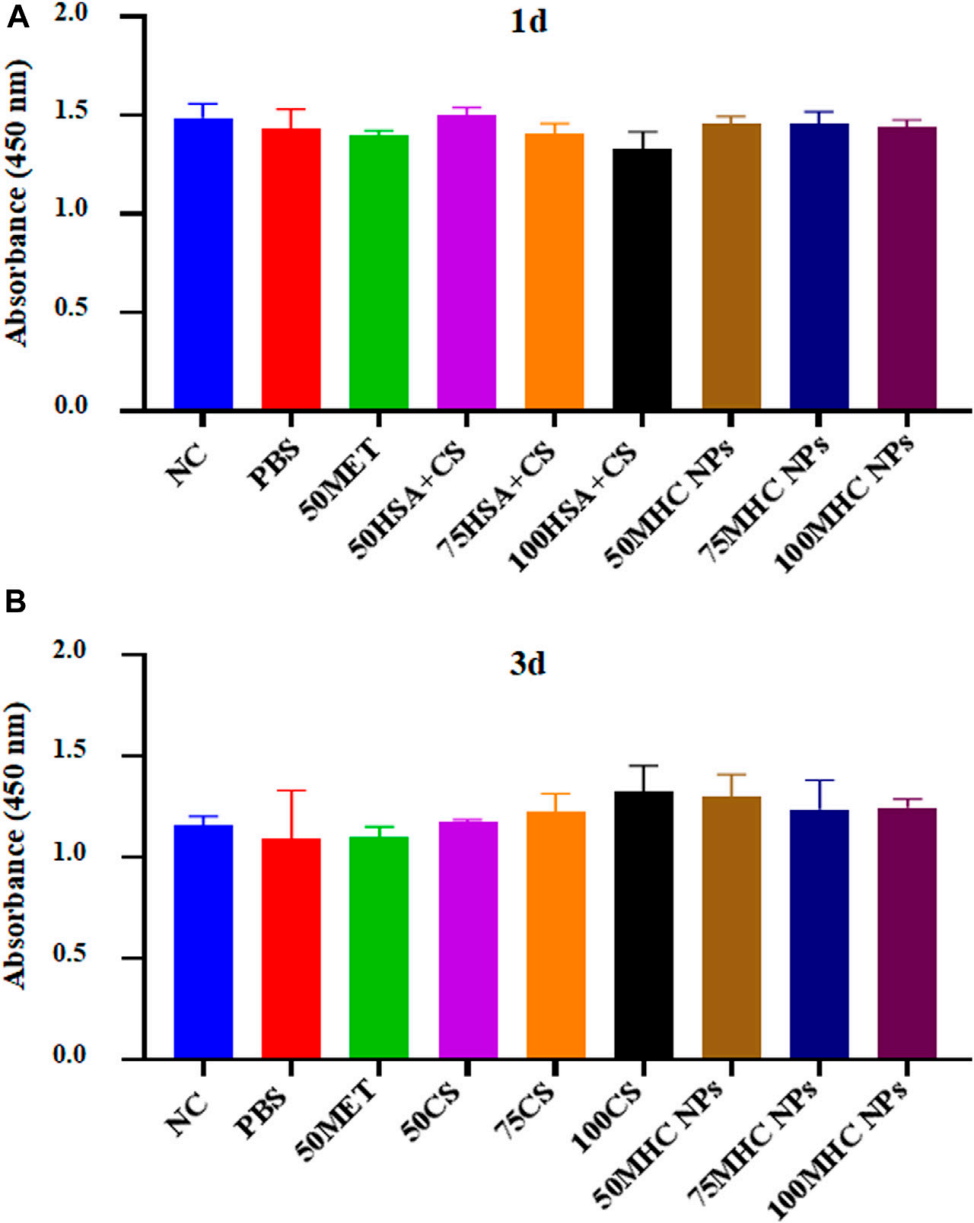
CCK-8 assay kit was used to analyze the cytotoxicity of the MHC NPs on BMSCs in 1st day **(A)** and 3rd day **(B)**. HSA, human serum albumin; MHC NPs, metformin/HSA/chitosan nanoparticles; CS, chitosan; MET, metformin; BMSCs, mice bone marrow mesenchymal stem cells.

Figure 7 shows BMSC proliferation on different concentrations of MHC NPs in 1st day and 3rd day compared to metformin and HSA + CS. Cell proliferation did not differ significantly between the groups.

### 3.4 Osteogenesis *in vitro* of MHC NPs

**FIGURE 8:**
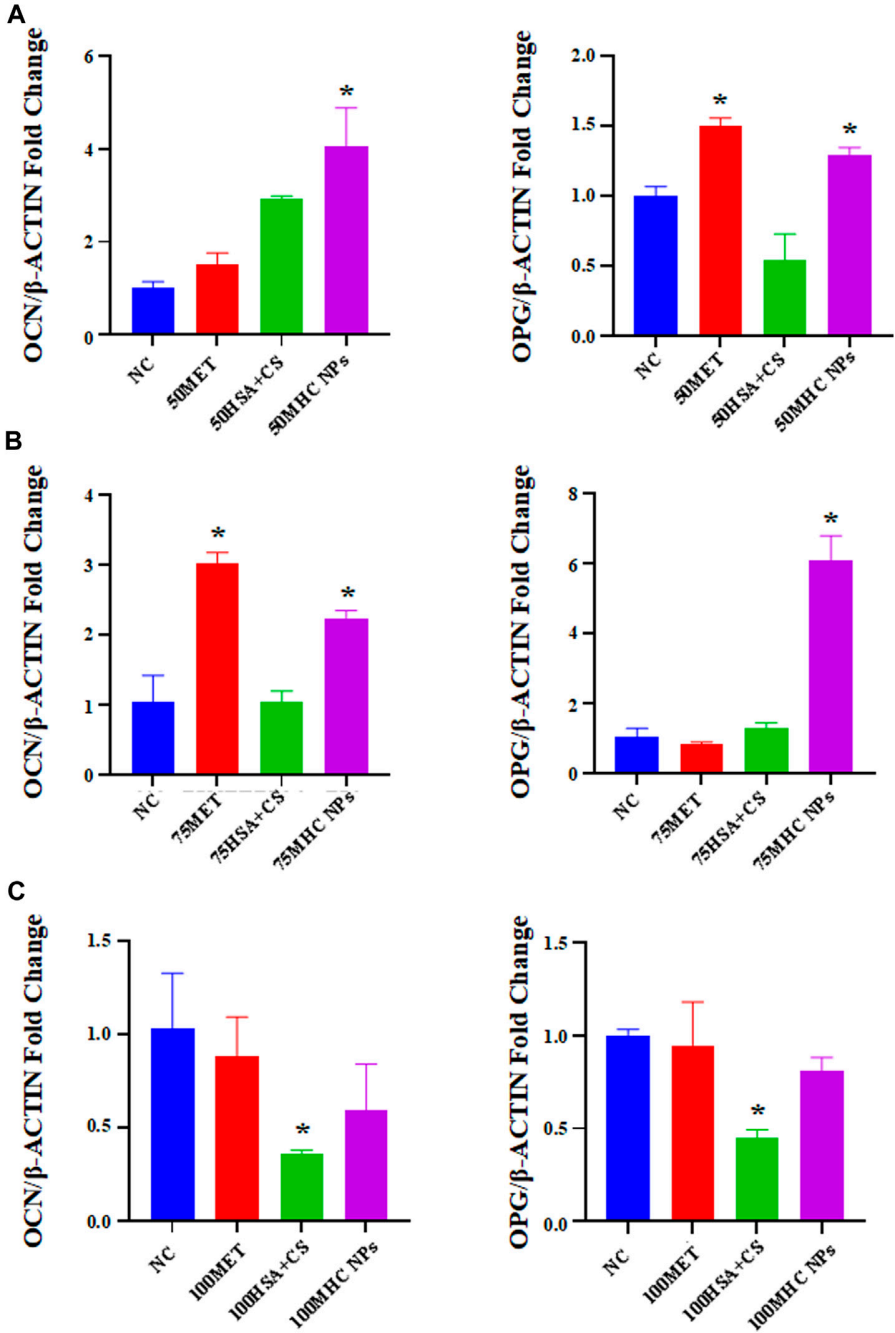
Gene expression of osteoblastic-related genes was analyzed by real-time PCR analysis. **(A)** Real-time PCR analysis showed that OCN expression in MHC NPs (50 μM) was higher than other groups, and OPG expression in MHC NPs group was higher than control group while lower than metformin group (*p* < 0.05). **(B)** OCN expression in metformin group (75 μM) was higher than other groups, and OPG expression in MHC NPs was higher than other groups (*p* < 0.05). **(C)** OPG and OCN expression in MHC NPs (100 μM) were not obvious and OPG and OCN expression in HSA + CS were lower than control group (*p* < 0.05). HSA, human serum albumin; MHC NPs metformin/HSA/chitosan nanoparticles; CS, chitosan; MET, metformin; BMSCs, bone marrow mesenchymal stem cells.

**FIGURE 9:**
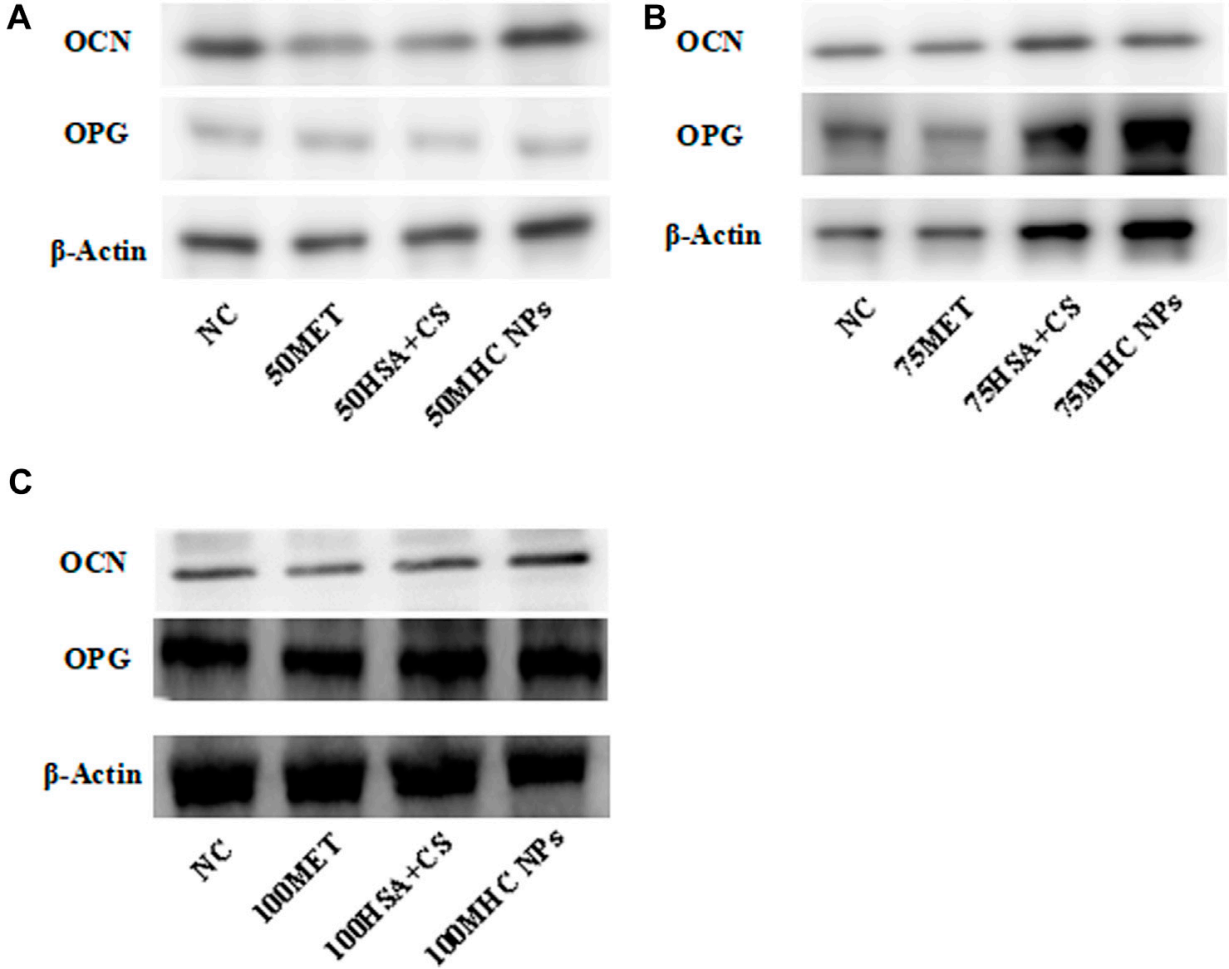
Western blot. **(A)** Under 50 μM concentration, 50 μM MHC NPs showed increased OCN and OPG expression. **(B)** Under 75 μM concentration, 75 μM MHC NPs showed increased OPG expression. **(C)** Under 100 μM concentration, OCN and OPG expression present no obvious difference. HSA, human serum albumin; MHC NPs, metformin/HSA/chitosan nanoparticles; CS, chitosan; MET, metformin.

**FIGURE 10:**
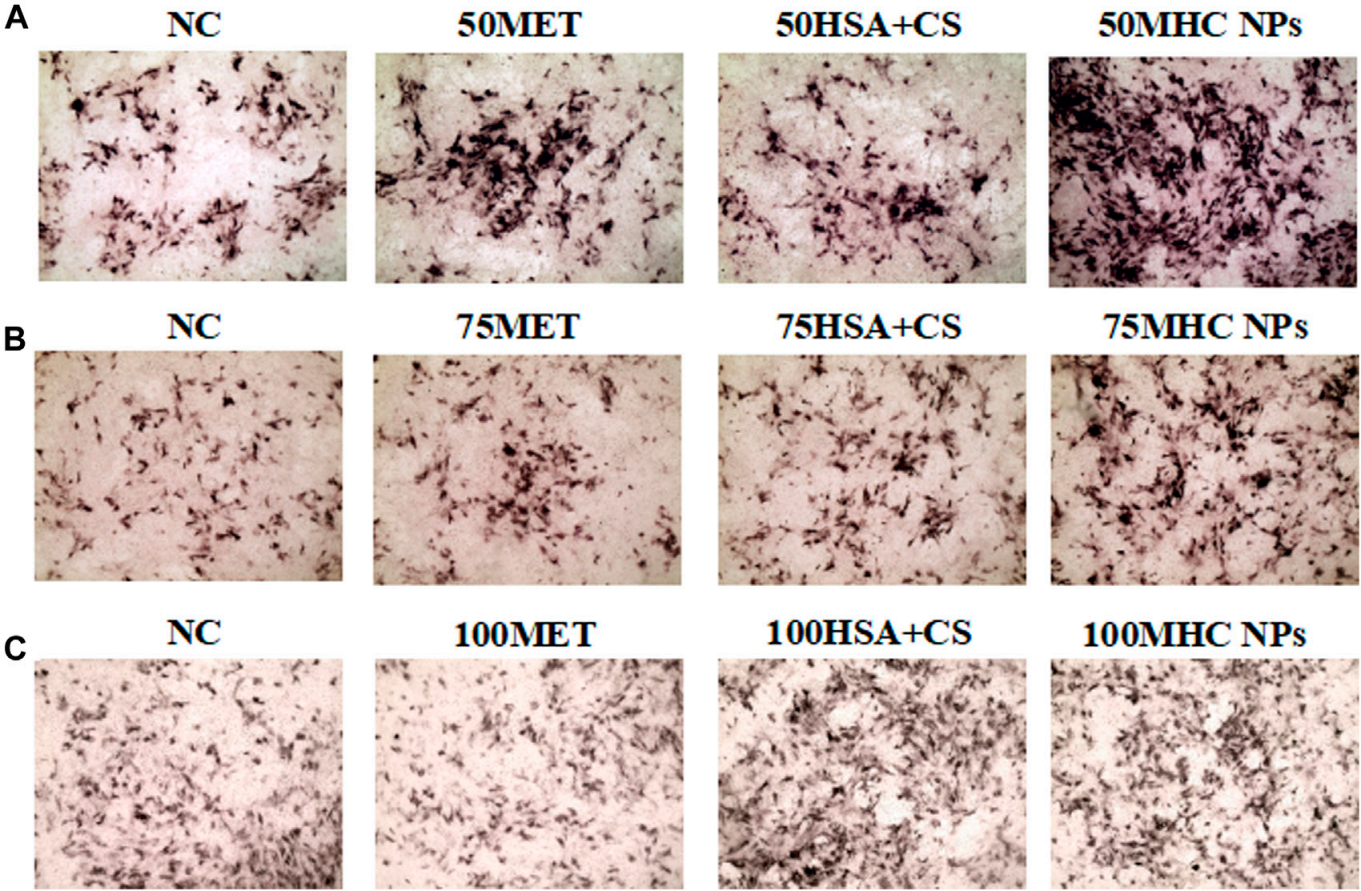
Osteogenic induction under MHC NPs at 50 μM **(A)**, 75 μM **(B)**, and 100 μM **(C)**, 50 and 75 μM MHC NPs could increase ALP expression (black, 4 ×). HSA, human serum albumin; MHC NPs, metformin/HSA/chitosan nanoparticles; CS, chitosan; MET, metformin.

At 50 μM concentration, real-time PCR analysis showed that OCN expression in MHC NPs was higher than that in other groups, and OPG expression in the MHC NPs group was higher than that in the control group and lower than that in the metformin group (*p* < 0.05, Figure 8A). Western blot analysis revealed that OCN and OPG were expressed at higher levels in the MHC NPs group (Figure 9A). It was found that 50 μM MHC NPs had the strongest effect on ALP staining (Figure 10A).

At 75 μM, real-time PCR analysis showed that OCN expression in the metformin group was higher than that in the other groups, and OPG expression in the MHC NPs group was higher than that in the other groups (*p* < 0.05, Figure 8B). According to the western blot analysis, OPG was expressed at a higher level in the MHC NPs group (Figure 9B). ALP staining was strongest under the action of 75 μM MHC NPs (Figure 10B).

At 100 μM, real-time PCR analysis showed that OCN and OPG expression in MHC NPs was not clear (Figure 8C). Western blot analysis showed that OCN and OPG were not clearly expressed (Figure 9C). There was no significant difference in ALP staining (Figure 10C).

## 4 Discussion

Regeneration of bone is a complex and well-organized process, and bone regeneration is required in large amounts in complex clinical conditions (Dimitriou et al., 2011). It is promising to combine a material regimen with bone repair (Tan et al., 2021). MHC NPs were assembled by stirring HSA, metformin hydrochloride, and chitosan mixture in a magnetic field. The characteristic peak of metformin in FTIR results were not obvious, but they were not the conclusive result to measure MHC NPs. In normal saline (pH 7.4) solution, metformin was encapsulated by more than 90%, and the release curve fit the zero-order kinetics distribution and remained at approximately 50% after 48 h. The diameter of the synthesized MHC NPs was 50 nm. It was found that it had good stability, biocompatibility and biological activity. Some studies suggest that calcium silicate nanoparticles doped with Cu, which have a diameter of 50 nm can heal bone (Mabrouk et al., 2019). Others reported that 50 nm spherical silica nanoparticles promote the osteoblasts differentiation and suppress bone-resorbing osteoclasts (Weitzmann et al., 2015). Therefore, we believe MHC NPs can meet the requirements of osteogenesis. MHC NPs were not cytotoxic in a CCK-8 assay. Next, we examined whether metformin could enhance the osteogenic differentiation of BMSCs in chitosan hydrogel growth environments (Cai et al., 2022). Alkaline phosphatase activity in BMSCs was significantly increased after 3 days of treatment with the studied MHC NPs. Additionally, MHC NPs upregulated OPG and OCN expression in BMSCs treated with them.

Because of its safety and low cost, metformin is a widely used biguanide drug (Lv and Guo, 2020). There is evidence that metformin regulates glucose control and extends lifespan of patients (MacNeil et al., 2020). The multiple functions of metformin, including the osteogenic differentiation of stem cells, have been demonstrated to support bone formation (Ma et al., 2018; Zhao et al., 2020; Bahrambeigi et al., 2019). However, the effect of different concentrations of metformin on the proliferation of BMSCs is still not clear. Our results showed that 50 μM metformin had the strongest effect in promoting osteogenic differentiation. Lower concentrations of metformin inhibited the osteogenic differentiation of BMSCs, but higher concentrations of metformin inhibited it. As reported by Chunxia et al., metformin induces osteogenesis in rat bone marrow mesenchymal stem cells (rBMSCs) in a dose-dependent manner, which is consistent with our results. However, Ren reported that the optimal metformin concentration for promoting osteogenesis is 1 mg/mL (Qasim et al., 2019). It is generally believed that rBMSCs are more active and have better stemness. We suggest that BMSCs in rats are better able to resist the toxicity of high concentrations of metformin and thus better exert their osteogenic effects. Thus, metformin rapid dilution and burst release in the affected bone site should be avoided. It is important to offer optimal storage and a controlled drug release system.

In recent years, NPs have been synthesized and studied as potential drug delivery systems aimed at improving drug delivery efficiency and maintaining a stable concentration of metformin (Bhattarai et al., 2006). Chitosan is a natural biopolymer derived from chitin by deacetylation, and it was selected as the polymer layer due to its wide use in drug delivery applications (Fakhri et al., 2020). Chitosan is recognized as a highly biocompatible and biodegradable polymer (Hashemi et al., 2020; Gulati et al., 2012). Chitosan has been shown to enhance osteogenesis *in vitro* in numerous studies (Hashemi et al., 2020). In this study, HSA, metformin hydrochloride, and chitosan mixtures were magnetically stirred to complete the assembly of MHC NPs. This process was inspired by the self-assembly principle through the interaction of denature/renature and negative/positive charge passed to HSA/chitosan depending on the content of ethanol and the charging effect of chitosan/HSA (Peng et al., 2017). HSA is the most abundant endogenous protein in plasma, which makes HSA an ideal biomaterial for drug delivery of paclitaxel, platinum-based drugs and chlorine E6. Usually, drug molecules are absorbed on the hydrophobic domains of HSA molecules by hydrophobic interactions and then encapsulated in HSA nanoparticles by desolvation (Xu et al., 2021). Figure 3 shows that MHC NPs are structurally stable and have a suitable nanoparticle size, and the synthesis process does not change the structure of the loaded metformin. Furthermore, the encapsulation (%) of metformin was more than 90%, indicating a sufficient drug loading effect. To evaluate MHC NPs stability at room temperature, HPLC was used to determine the free metformin release from MHC NPs. The obtained results showed that MHC NPs exhibited good stability for 20 days at room temperature, indicating the stability of MHC NPs in normal saline.

We detected the concentration of chitosan metformin that promoted BMSCS osteogenesis CCK-8 intervention in BMSCs was usually 1–3 days, and osteogenic induction *in vitro* was usually 3–7 days (Chen et al., 2022). As ALP is an early osteogenic marker, significant differences in osteogenic differentiation ability were observed in different groups at 3rd.Therefore, we used 1-day and/or 3-day tests to demonstrate the effect of MHC NPs on bone formation in BMSCs. We found that 50 μM nanoparticles had the best osteogenic activity, which corresponded to our previous experimental results in Figure 3. We hypothesized that MHC NPs mainly promote bone formation through the osteogenesis of metformin and the anti-inflammatory effect of chitosan. Metformin promotes osteogenic differentiation of BMSCs partly by inhibiting the activity of GSK3β (Ma et al., 2018). Metformin can regulate the expression of proangiogenic/osteogenic growth factors and osteoclasts in SHEDs and induce their osteogenic differentiation by activating the AMPK pathway (Zhao et al., 2020). Studies have shown that chitosan inhibits LPS-induced inflammatory responses in macrophages, including the expression and release of proinflammatory mediators. These inflammatory mediators include tumor necrosis factor-α (TNF-α), interleukin-6 (IL6), inducible nitric oxide synthase (iNOS), cyclooxygenase-2 (COX-2), prostaglandin E2 (PGE2) and nitric oxide (NO) (Lee et al., 2009). COS has also been shown to reduce systemic inflammatory responses, as indicated by serum levels of TNF-α and IL-1β and damage to the liver, kidney, and lung in a mouse model of LPS-induced sepsis (Qiao et al., 2011). In addition, due to positively charged chitosan chains, chitosan can improve the growth, replication and cell-shape retention of osteoblasts to promote osteogenesis (Hashemi et al., 2020). However, the specific mechanism needs to be further studied.

In this work, we report the preparation and characteristics of a drug delivery system mixture by human serum albumin, metformin hydrochloride, and chitosan mixture to sustain the release of metformin. This may have implications for the development of medical devices that incorporate drugs.

## Data Availability

The raw data supporting the conclusion of this article will be made available by the authors, without undue reservation.
