# Supplementary figures and images for: Chitosan nanoparticles for sustained release of metformin and its derived synthetic biopolymer for bone regeneration
Source: Front Bioeng Biotechnol. 2023 Jul 5;11:1169496. doi: 10.3389/fbioe.2023.1169496 (PMC10354276; doi:10.3389/fbioe.2023.1169496)

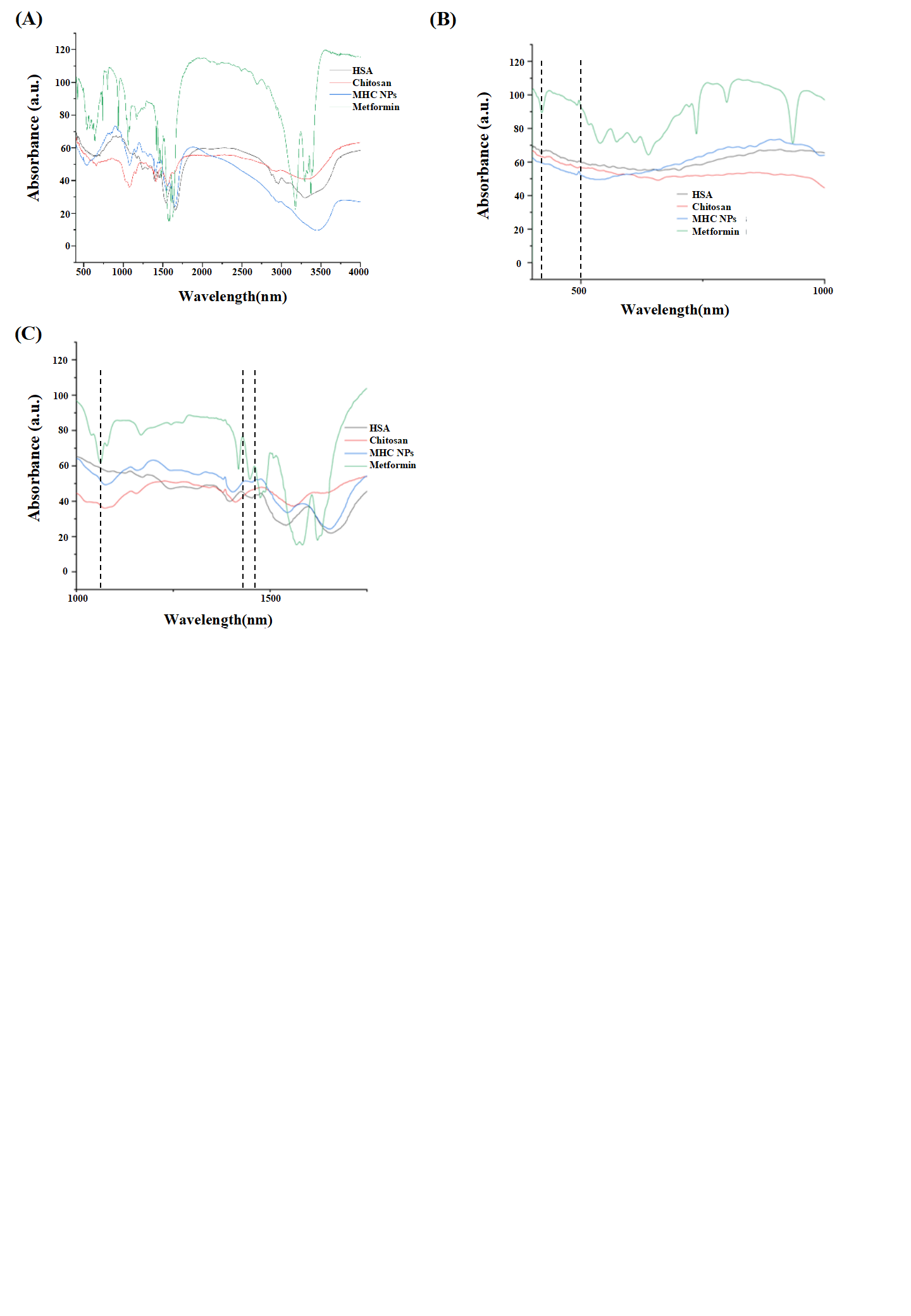

Supplement: Supplementary file 1 [file Image1.TIF]
